# Space‐Confined Synthesis of Sulfonated Covalent Organic Framework‒Polymer Membranes for Enhanced Osmotic Energy Conversion

**DOI:** 10.1002/smll.202508217

**Published:** 2025-08-25

**Authors:** Yumeng Guo, Xiang Sun, Qianxi Zhang, Ze‐Xian Low, Huanting Wang, Ying Zhu, Lei Jiang

**Affiliations:** ^1^ State Key Laboratory of Materials‐Oriented Chemical Engineering National Engineering Research Center for Special Separation Membrane Nanjing Tech University Nanjing China; ^2^ Department of Chemical and Biological Engineering Monash University Clayton Victoria 3800 Australia; ^3^ Key Laboratory of Bio‐Inspired Smart Interfacial Science and Technology Ministry of Education School of Chemistry Beihang University Beijing 100191 China; ^4^ Beijing Advanced Innovation Center for Biomedical Engineering Beihang University Beijing 100191 China; ^5^ CAS Key Laboratory of Bio‐inspired Materials and Interfacial Science Technical Institute of Physics and Chemistry Chinese Academy of Sciences Beijing 100190 China

**Keywords:** covalent organic framework, nanofluidic membrane, osmotic energy conversion, polymer membrane

## Abstract

Osmotic energy, an infinite, clean energy source, can be efficiently harnessed through reverse electrodialysis using ion‐selective membranes. While polymeric membranes are excellent candidates due to their solution‐processability and scalability, their non‐uniform pore architecture and high resistance limit their power density output. Here, an in situ space‐confined synthesis strategy is proposed to fabricate sulfonated covalent organic frameworks within a sulfonated polymeric network, resulting in interconnected, well‐defined ion channels. This allows a maximum power density reaching up to 40.33 W m^−2^ under a 500‐fold salinity gradient and a real‐world power density of 14.84 W m^−2^ when extracting osmotic energy from natural seawater and river water. This study underscores the potential of space‐confined synthesis strategies in creating flexible and scalable ion‐selective membranes for efficient salinity gradient energy harvesting, marking a significant step toward their practical applications.

## Graphical abstract

A sulfonated covalent organic framework within a sulfonated polymeric network, resulting in interconnected, well‐defined ion channels is fabricated by an in‐situ space‐confined synthesis strategy. This allows a maximum power density reaching up to 40.33 W m^−2^ under a 500‐fold salinity gradient and a real‐world power density of 14.84 W m^−2^ when extracting osmotic energy from natural seawater and river water.

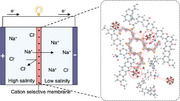

## 1 Introduction

The scarcity of traditional energy resources, coupled with rising energy demands, has spurred significant interest in harnessing energy from the difference in salt concentration between seawater and freshwater.^[^ ^1^, ^2^, ^3^ ^]^ Among the various technologies designed to harness this energy, reverse electrodialysis (RED) stands out for its ability to directly convert the Gibbs free energy difference into electrical power.^[^ ^4^, ^5^ ^]^ In RED systems, ion‐selective membranes (ISMs) play a crucial role by allowing the selective passage of specific ions while blocking those of opposite charge, leading to charge accumulation and the creation of a transmembrane potential.^[^ ^6^, ^7^ ^]^ Consequently, improving the selective ion passage and ion transport rate of ISMs is essential for achieving high power densities in RED systems.^[^ ^8^ ^]^

Conventional ISMs, such as ion exchange membranes, rely on fixed‐charged groups to facilitate selective ion transport.^[^ ^9^, ^10^, ^11^ ^]^ However, these membranes often suffer from limited ion transport due to structural factors such as channel tortuosity and narrow pathways, which collectively diminish the output power density output.^[^ ^2^, ^12^, ^13^, ^14^ ^]^ To maximize power generation, an ideal ISM should feature a high density of ion channels with optimal sizes to promote efficient ion transport. Furthermore, these channels should present minimal energy barriers, allowing counterions to permeate freely across the membrane.^[^ ^15^, ^16^, ^17^ ^]^ Incorporating porous frameworks into polymer membranes has emerged as an effective strategy to introduce more channels with suitable dimensions, enhancing ion transport.^[^ ^18^, ^19^, ^20^ ^]^ Despite this, challenges such as filler agglomeration and incompatibility with the polymer matrix can create non‐selective voids, limiting the membrane's performance.

Here, we present a space‐confined synthesis (SCS) strategy to in situ synthesize sulfonated‐covalent organic frameworks (COFs) within sulfonated poly(ether ether ketone) (SPEEK) matrix, forming nanofluidic membranes that overcome the geometric constraints of traditional polymer membranes and enhance power density output. This bottom‐up approach improves the compatibility between the COFs and the SPEEK polymer matrix, resulting in highly permeable membranes with enhanced ion selectivity and flux, as well as an easily scalable fabrication process. The sulfonated‐COFs (TpPa‐SO_3_H), characterized by abundant negative charges and an intrinsic porous framework, ensure efficient selective ion transport and increased ion permeation. Using the SPEEK/SCOF nanocomposite membrane (SCM) to extract osmotic energy between natural seawater and river water, we achieved a notably high power density of 14.84 W m^−2^.

## 2 Results and Discussion

2.1

**Figure 1:**
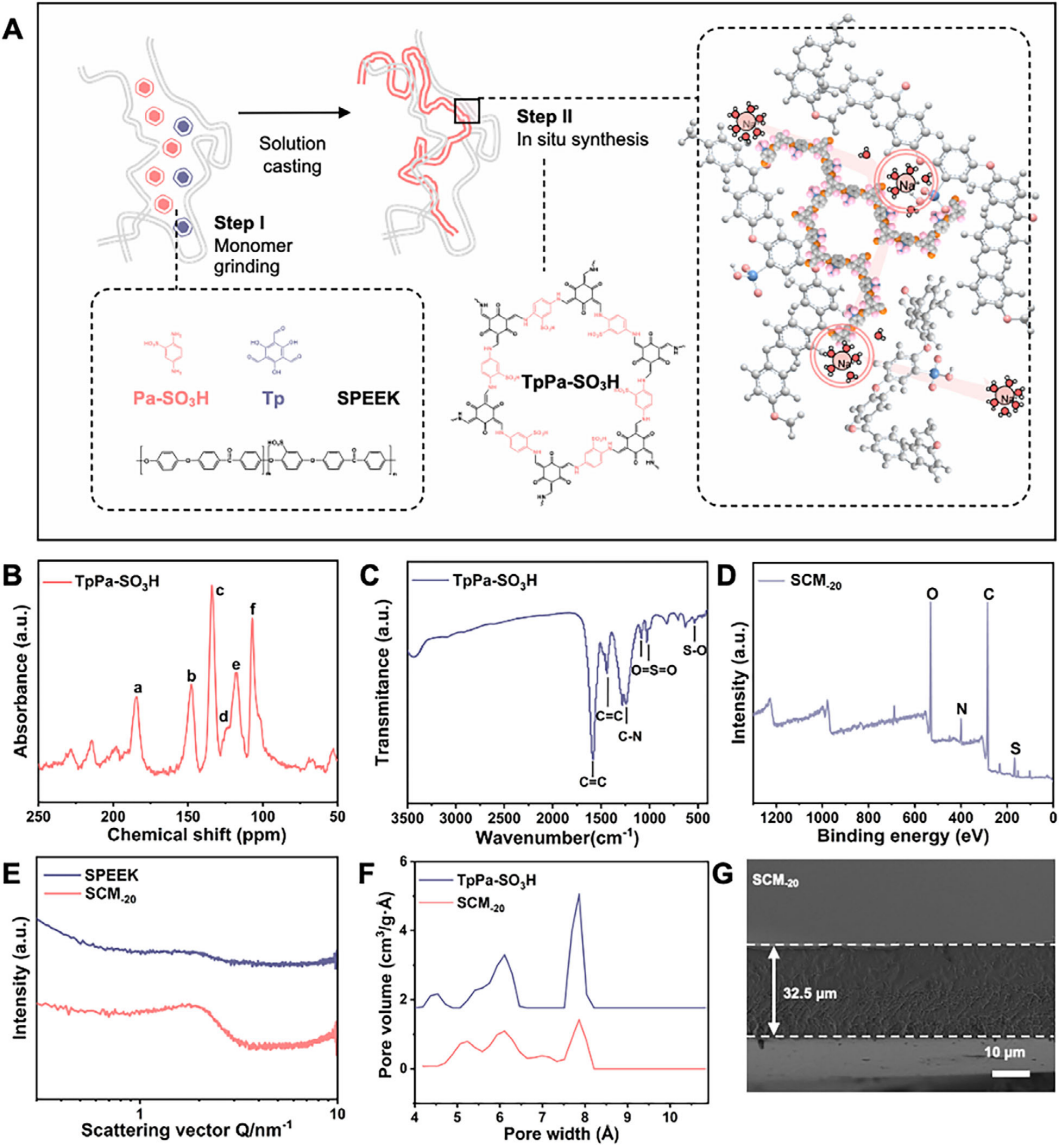
Fabrication and characterization of SCM. A) SCS strategy. B) Solid‐state carbon‐13 nuclear magnetic resonance (^13^C NMR) spectra of TpPa‐SO_3_H. C) FTIR absorption bands of TpPa‐SO_3_H. D) XPS survey spectra. E) SAXS patterns of SPEEK and SCM_‐20_. F) Pore size distributions derived via density functional theory (DFT) modeling based on adsorption isotherm data for TpPa‐SO_3_H and SCM_‐20_. G) Cross‐sectional micrograph of SCM_‐20_.

**Figure** **1**A illustrates the SCS strategy of SCM. First, we ground the two COF monomers (Tp and Pa‐SO_3_H) along with the catalyst (PTSA) in a mortar, then slowly added DMSO and SPEEK (sulfonation degrees of 75.5%, Figure S1, Supporting Information) dope solution to the mixture, causing the dope solution to change from yellow to red (Step I). A homogeneous dope solution containing COF monomers and SPEEK was uniformly coated onto a glass substrate via a blade‐coating technique. Subsequently, the films were heated to induce the in situ formation of COFs within the SPEEK membrane, ultimately yielding the SCM composite membrane (Step II; see Figure S2 and Methods for detailed steps, Supporting Information).

To investigate the in situ synthesis of TpPa‐SO_3_H within the SPEEK membrane, we redissolved the SCM in DMSO and separated the pellet by centrifugation. ^13^C NMR confirmed the formation of the COF, resonances observed between 107 and 134 ppm are assigned to aromatic carbons within benzene rings. Signals detected at 152, 179, and 187 ppm are ascribed to the conjugated C═C and carbonyl (─C═O) functionalities, confirming the formation of keto‐enamine linkages, which aligns with prior literature (Figure 1B).^[^ ^21^ ^]^ Fourier‐transform infrared (FTIR) analysis of TpPa‐SO_3_H (Figure 1C) reveals distinct absorption bands at 1084 and 1027 cm^−1^, linked to asymmetric stretching vibrations of sulfonic acid O═S═O groups and aromatic ring vibrations in the TpPa‐SO_3_H framework, respectively. Additional peaks at 1582 and 1241 cm^−1^, attributed to C═C and C─N stretching modes, validate the enol‐keto tautomerization process.^[^ ^22^ ^]^ The absence of characteristic C═O stretching (1644 cm^−1^) from Tp monomers and N‐H stretching (3300–3425 cm^−1^) from Pa‐SO_3_H in the composite spectrum (Figure S3, Supporting Information) confirms successful covalent assembly of TpPa‐SO_3_H.^[^ ^23^ ^]^ X‐ray diffraction (XRD) patterns (Figure S4, Supporting Information) validate the effective incorporation of TpPa‐SO_3_H into the SPEEK matrix, as evidenced by distinct Bragg reflections at 5.1° and 26.4° in SCM_‐20_. X‐ray photoelectron spectroscopy (XPS) survey spectra (Figure 1D) provide additional confirmation of TpPa‐SO_3_H integration, displaying prominent C 1s, N 1s, O 1s, and S 2p core‐level signals. Small‐angle X‐ray scattering (SAXS) results (Figure 1E) indicate that the peaks for the SPEEK membrane and the SCM are positioned nearly identically, suggesting that the in situ synthesis strategy of TpPa‐SO_3_H within the SPEEK matrix does not significantly alter the existing nanostructure. This finding implies that TpPa‐SO_3_H is well‐dispersed within the polymer matrix, maintaining the inherent nanostructure of the SPEEK membrane. Furthermore, CO_2_ adsorption/desorption isotherm data, as seen in the pore size distribution (Figure 1F), demonstrating comparable pore dimensions between SCM_‐20_ and TpPa‐SO_3_H. Scanning electron microscopy (SEM) analysis indicated that SCM surfaces exhibited dense microstructures, with surface roughness increasing with higher TpPa‐SO_3_H content (Figure S5, Supporting Information). Atomic force microscopy (AFM) imaging further revealed that both upper and lower surfaces of SCM_‐20_ displayed morphological characteristics closely resembling those of the SPEEK membrane (Figure S6, Supporting Information). Figure S7 (Supporting Information) displays the morphology of TpPa‐SO_3_H recovered from SCM_‐20_, which are smaller in size compared to directly synthesized TpPa‐SO_3_H (Figure S8, Supporting Information). The cross‐sectional image of SCM_‐20_ exhibited a dense and continuous morphology (Figure 1G). Energy‐dispersive X‐ray (EDX) elemental mapping (Figure S9, Supporting Information) confirmed homogeneous dispersion of constituent elements (S, C, N, O) of TpPa‐SO_3_H network within the SPEEK membrane. In contrast, the pure SPEEK membrane (Figure S10, Supporting Information) showed no N content. These results confirmed the successful in situ synthesis of TpPa‐SO_3_H in the SCM. In addition, the introduction of TpPa‐SO_3_H enhanced the hydrophilicity of SCM_‐20_ (Figure S11, Supporting Information). The optimized SCM composition achieves an effective balance between pore density and mechanical integrity. While increasing COF content enhances porosity, it reduces mechanical strength due to decreased polymer packing density (Figure S12, Supporting Information). Our composite design overcomes this trade‐off through optimal material selection and processing, maintaining both robust mechanical properties (sufficient for RED applications) and high porosity.

### 2.2 Ion Transport Behaviors of SCM_‐20_ and SPEEK

**Figure 2:**
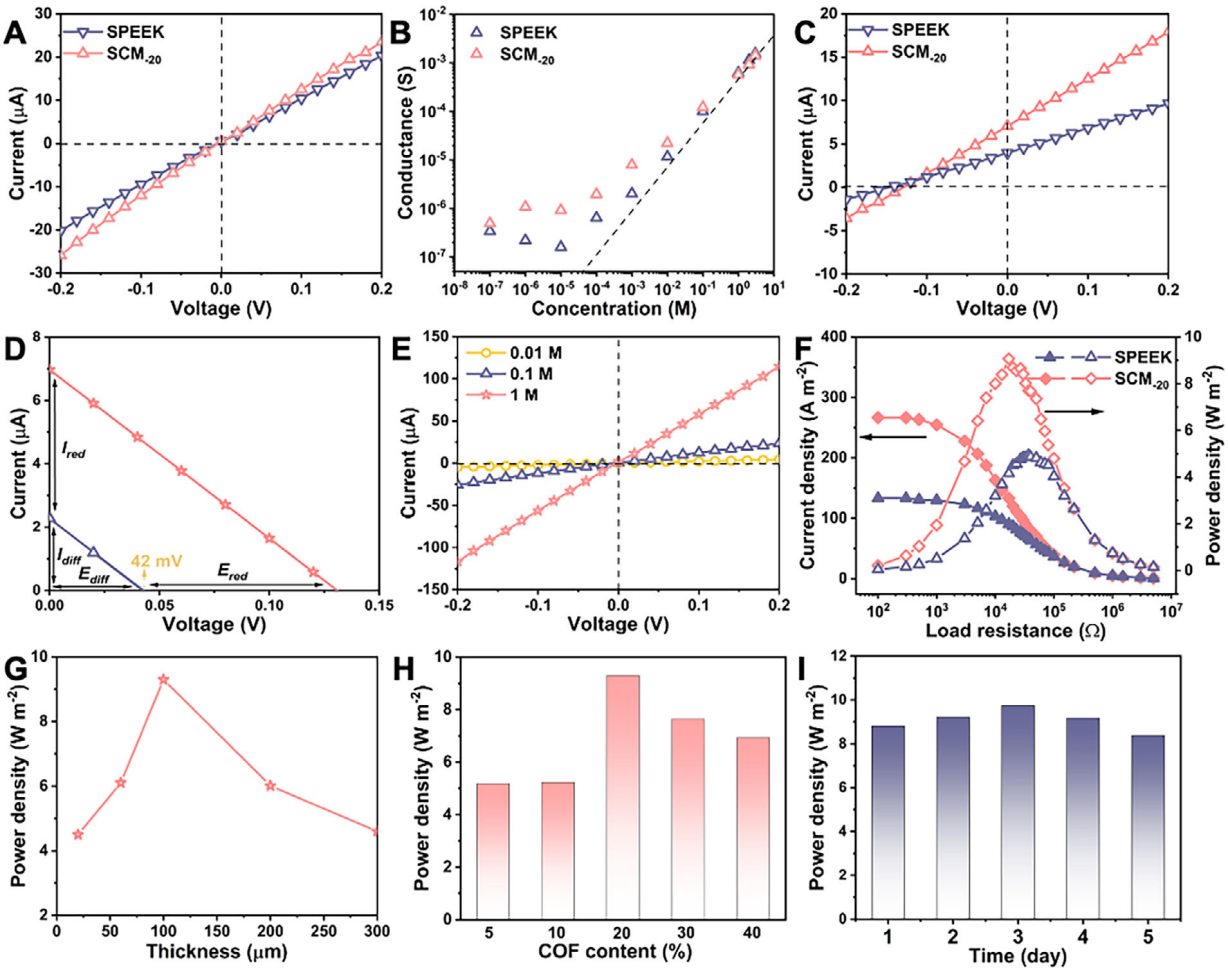
Ion transport performance and salinity‐gradient energy harvesting characteristics of SPEEK and SCM_‐20_ membranes. A) *I–V* profiles of SPEEK and SCM_‐20_ in 0.1 m KCl. The curves exhibited symmetry across the potential range of −0.2 to 0.2 V. B) Concentration‐dependent ionic conductance of SPEEK and SCM_‐20_ in KCl solutions revealed deviations from bulk electrolyte behavior at low concentrations, suggesting surface charge‐mediated ion transport regulation. C) Under a 50‐fold salinity gradient, *I–V* curves of SPEEK and SCM_‐20_ membranes yielded *V_OC_* and *I_SC_* values derived from coordinate axis intercepts. D) *I–V* characteristics under a 10 mm/0.5 m concentration gradient (cap side containing the high‐concentration solution) demonstrated distinct transport behavior. E) Representative *I–V* responses of SCM_‐20_ were recorded across varying KCl concentrations. F) Comparative power density analysis under 50‐fold salinity gradients showed SCM achieving peak performance when external load resistance matched internal membrane resistance. G) Thickness‐dependent power density trends of SCM membranes and H) COF content optimization studies (50‐fold NaCl gradient) identified maximum efficiency thresholds. I) Long‐term operational stability of SCM_‐20_.

To probe ionic dynamics within the SPEEK matrix, transmembrane ion flux was analyzed using a custom electrochemical setup (Figure S13, Supporting Information). Current‐voltage (*I–V*) curves of SPEEK and SCM_‐20_ membranes were acquired in 0.1 M KCl electrolyte under a voltage sweep from −0.2 to +0.2 V (**Figure** **2**A). The SCM_‐20_ exhibited significantly enhanced ion transport efficiency compared to pristine SPEEK. Concentration‐dependent conductance profiles were subsequently measured across varying KCl concentrations. At dilute regimes, conductance deviated from bulk solution behavior, implying surface‐charge‐dominated ion migration (Figure 2B). Ion selectivity analysis was performed by establishing a transmembrane gradient (1 m KCl vs 0.01 m KCl). The minimal contribution of the low‐concentration reservoir to the net current allowed dominant ion flux from the high‐concentration side. As shown in Figure S14 (Supporting Information), K⁺‐driven current substantially exceeded Cl^−^‐mediated transport, confirming cation‐selective behavior in SCM_‐20_. Under a 50‐fold NaCl concentration gradient, spontaneous ion diffusion generated measurable open‐circuit voltage (*V_OC_*) and short‐circuit current (*I_SC_*), attributed to Gibbs free energy disparities.^[^ ^24^, ^25^ ^]^ Selective cation/anion permeation through the membrane induced charge separation, yielding *I_SC_* and *V_OC_*. For SCM_‐20_, *I_SC_* reached 7.06 µA (Figure 2C), 78% higher than SPEEK (3.96 µA), consistent with enhanced surface charge density from TpPa‐SO_3_H incorporation. Ion selectivity coefficients (S) were quantified using established models^[^ ^26^ ^]^ where S = 1 denotes ideal selectivity and S = 0 indicates non‐selective transport. It can be calculated using the following equation:

(1) $$s=\frac{E_{diff}}{\frac{RT}{F}\ln\left(\frac{\gamma_{NaCl}^{c_{H}}}{\gamma_{NaCl}^{c_{L}}}\right)}$$

where *R* (universal gas constant), *T* (temperature), *F* (Faraday constant), and $\gamma_{NaCl}^{c_{H}}$ and $\gamma_{NaCl}^{c_{L}}$ (Na⁺ activity coefficients in high/low‐concentration solutions) define the thermodynamic driving forces. Under a 50‐fold concentration gradient, SCM_‐20_ exhibited a *V_OC_* of 128 mV and an *I_SC_* of 7.06 µA, extracted from *I–V* curve analysis (Figure 2D). After correcting for redox potential contributions from the base window, the net *V_OC_* attributable to nanochannel‐driven diffusion reached ≈86 mV. The derived ion selectivity coefficient (*S*) ≈0.92, reflecting near‐ideal cation selectivity, a critical feature for efficient energy harvesting. As illustrated in Figure S15 (Supporting Information), both *V_OC_* and *I_SC_* scaled monotonically with increasing salinity gradients, achieving maxima of 169 mV and 367 A·m^−2^, respectively. When application of external bias across the concentration gradient (Figure 2E) produced linear current‐voltage responses, confirming dominant ohmic transport mechanisms.

### 2.3 Salinity‐Gradient Energy Harvesting of SPEEK and SCM_‐20_

A RED system was implemented with Ag/AgCl electrodes to investigate the osmotic energy harvesting potential of SCM_‐20_ under a 50‐fold NaCl gradient, incorporating an external load resistance (*R_L_*) (Figure S16, Supporting Information). The *I–V* characteristics of SCM_‐20_ under this gradient (Figure S17, Supporting Information) displayed comparable profiles for both forward and reverse ion diffusion directions, confirming structural symmetry and absence of directional bias in ion transport. The intercept parity on the voltage and current axes further validated this equilibrium. As illustrated in Figure 2F, the maximum power density of SCM_‐20_ (9.3 W m^−2^), substantially outperforms the pristine SPEEK (4.93 W·m^−2^). Thickness‐dependent measurements (Figure 2G) identified optimal performance at a blade‐coated membrane thickness of 100 µm. Furthermore, increasing TpPa‐SO_3_H content in SCM membranes enhanced power density, peaking at 20 wt.% COF loading (Figure 2H). This enhancement can be attributed to two factors: a well‐connected COF network enhances ion conductivity and selectivity, whereas excessive COF content may lead to aggregation within the membrane, hindering ion transport.

Since seawater is composed of various dissolved cations, including Na^+^, Ca^2+^, K^+^, and Mg^2+^ ions, we analyzed how cation type alters ionic current dynamics and the osmotic energy output of SCM_‐20_. As shown in Figure S18 (Supporting Information), the power densities obtained with LiCl, NaCl, KCl, CaCl_2_, MgCl_2,_ and AlCl_3_ were 5.29, 9.3, 12, 10, 7.4, and 5.71 W m^−2^, respectively, highlighting its potential for hypersaline energy harvesting across diverse electrolytes. Notably, the SCM_‐20_ membrane achieved power densities of 9.3 W·m^−2^ (SCM_‐20_) and 4.93 W·m^−2^ (SPEEK), substantially exceeding prior benchmarks,^[^ ^27^ ^]^ For comparison, pure TpPa‐SO_3_H COF membranes typically yield ≈5.9 W·m^−2^,^[^ ^23^ ^]^ highlighting the enhanced performance of our structurally engineered composite. This performance enhancement arises from the complementary advantages of both components: SPEEK provides excellent Na⁺ transport through its tunable sulfonated nanochannels, while the COF framework contributes ordered pores and stable charge distribution. Moreover, the higher valence of divalent cations (Ca^2^⁺) enabled equivalent ionic current generation with half the molar flux compared to monovalent ions (Li⁺), thereby amplifying power density in CaCl_2_ systems relative to LiCl. This aligns with established cation valence‐dependent energy conversion mechanisms.^[^ ^24^, ^28^ ^]^ SCM_‐20_ exhibited robust osmotic energy harvesting performance with stable operation over 5 days (Figure 2I). Observed power density variations (<5%) correlated directly with electrolyte concentration equilibration dynamics.

**Figure 3:**
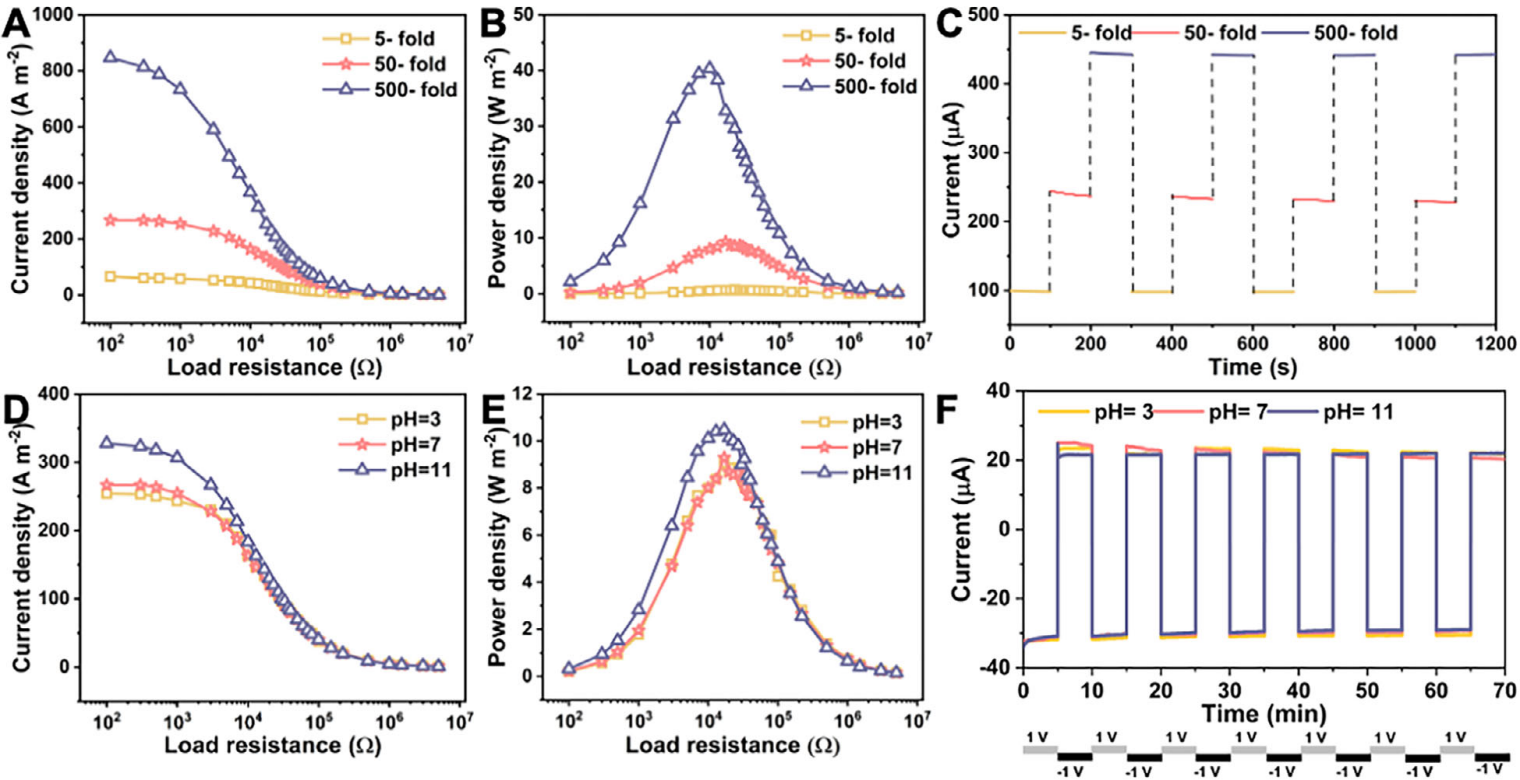
Environmental robustness of SCM_‐20_. A) Current density and B) power density profiles of SCM_‐20_ measured across varying concentration gradients, illustrating enhanced performance with increasing concentration differences. C) Current‐time (*I–T*) under cyclic concentration gradients, demonstrating excellent reversibility and stability over multiple cycles. D) Current density and E) power density measurements across a broad pH spectrum, highlighting consistent functionality under chemically diverse conditions. F) Current‐time (*I–T*) profiles in 0.1 m KCl under alternating voltage bias, showing SCM_‐20_ robust resistance to acidic and alkaline environments.

To comprehensively evaluate the osmotic energy conversion capabilities of SCM_‐20_, its environmental robustness was systematically examined. As demonstrated in **Figure** **3**A, current density exhibited a monotonic increase with escalating salinity gradients. Remarkably, under a 500‐fold gradient, SCM_‐20_ demonstrated exceptional performance with a power density of 40.33 W·m^−2^ (Figure 3B), surpassing conventional ion‐exchange membranes. Additionally, repeated current density measurements across multiple gradient cycles demonstrated excellent reversibility and stability over multiple cycles (Figure 3C). pH stability is critical for practical applications of ion‐selective membranes, as performance can be significantly affected by acidic and alkaline environments. As shown in Figure 3D,E, a marginal improvement in current density and power output was observed with rising pH, potentially linked to modulated surface charge or ion transport dynamics. Moreover, Figure 3F demonstrates that SCM_‐20_ exhibits excellent resistance to both acidic and alkaline conditions. Overall, SCM_‐20_ maintained stable performance under various concentration gradients and across a range of pH environments.

### 2.4 Molecular Dynamics Simulation of Na^+^ Ion Transport in SCM_‐20_ and SPEEK

**Figure 4:**
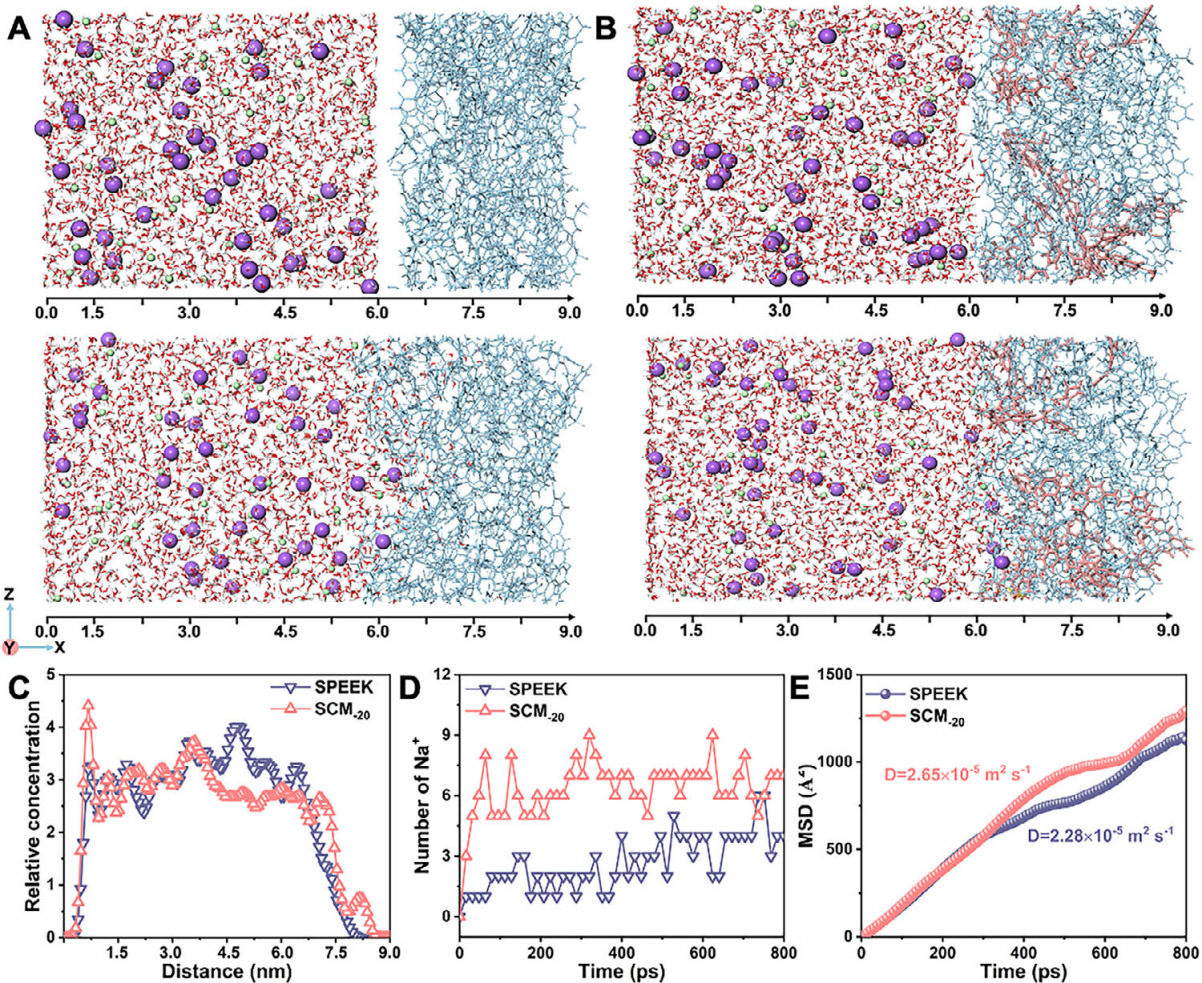
Dynamic equilibrium process of Na^+^ ions in SPEEK and SCM models. A) A snapshot of SPEEK membrane at 0 and 800 ps during the MD simulation. B) A snapshot of SCM at 0 and 800 ps during the MD simulation. C) Relative concentration distribution of Na^+^ ions inside the SPEEK and SCM membranes at different times. D) Number of Na^+^ ions passing through the SPEEK and SCM membranes. E) MSD profiles of Na^+^ ions in SPEEK and SCM, illustrating differences in ion mobility.

We performed molecular dynamics simulations to elucidate the ion transport mechanism governed by sulfonic acid groups in SPEEK and SCM_‐20_. The molecular structures of SPEEK and SCM_‐20_ were constructed for simulation calculations (Figure S19, Supporting Information), and **Figure** **4**A,B present snapshots of SPEEK and SCM_‐20_ at t = 0 and 800 ps during the simulation runs, respectively, using specific parameters (membrane thickness = 3 nm and NaCl = 6 nm). A high concentration of NaCl (C_H_ = 0.5 m) was introduced on one side of the membrane to diffuse across into pure water on the opposite side. Figure 4C depicts the ion concentration distribution along the *X*‐axis (001 direction), showing that the ions penetrate almost the entire length of the simulation box. Detailed analysis of the 6–9 nm region revealed that, although a higher Na^+^ ion concentration accumulated around the surface of the SPEEK membrane compared to the SCM_‐20_ surface, greater Na^+^ ion transport occurred within SCM_‐20_. This indicates that Na^+^ ions traverse the SCM_‐20_ more rapidly than through the SPEEK membrane. Additionally, as shown in Figure 4D, there was a marked discrepancy in the number of Na^+^ ions passing through SPEEK and SCM_‐20_ over time. At 800 ps, the number of Na^+^ ions passing through SCM_‐20_ was substantially higher than that through SPEEK. To investigate diffusion behavior of Na^+^ ions, we calculated the mean square displacement (MSD) and self‐diffusion coefficient (D) in the x‐direction, which describe the motion of the Na^+^ ions within the membranes. Figure 4E shows that the MSD and self‐diffusion coefficient of Na^+^ ions in the SPEEK membrane (D = 2.28 × 10^−5^ m^2^ s^−1^) were lower than those in SCM_‐20_ (D = 2.65 × 10^−5^ m^2^ s^−1^). This difference in self‐diffusion coefficients indicates enhanced ion transport in SCM_‐20_. The simulation results confirm the synergistic contribution of COF and SPEEK to the superior osmotic energy output of SCM_‐20_.

### 2.5 Real‐World Energy Harvesting Performance

**Figure 5:**
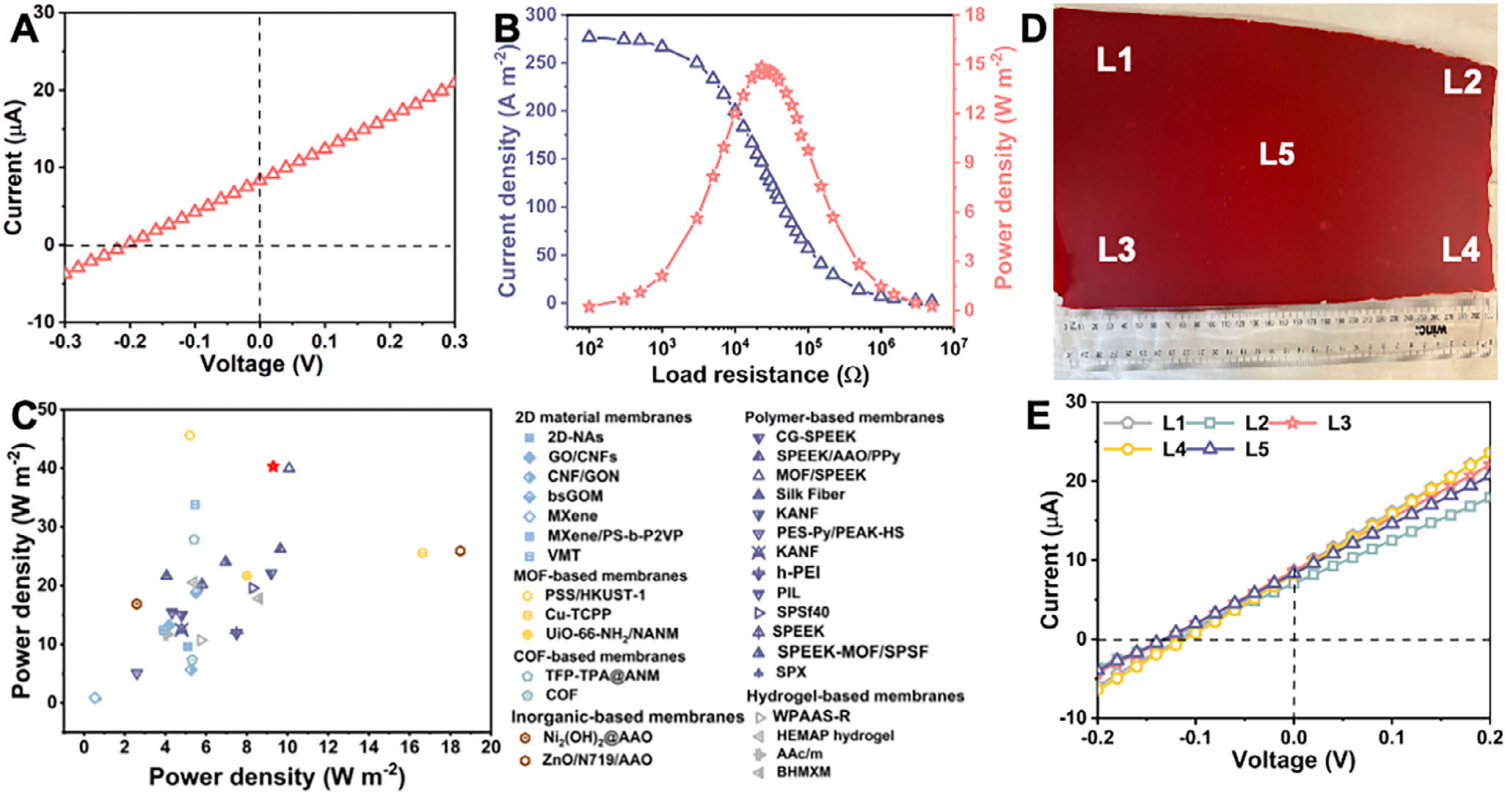
Osmotic energy conversion performance using natural water sources. A) The *I–V* characteristics of SCM_‐20_ under natural seawater/river water conditions. B) Current density and power density outputs of SCM_‐20_ are achieved through salinity gradient mixing of natural water sources. C) Comparison of salinity‐gradient energy conversion performance of SCM_‐20_ with previously reported membranes, highlighting its output power density under 50 (*X*‐axis) and 500‐fold (*Y*‐axis) NaCl concentration gradients (a line is provided to guide the eye).^[^ ^24^, ^28^, ^29^, ^30^, ^31^, ^32^, ^33^, ^34^, ^35^, ^36^, ^37^, ^38^, ^39^, ^40^, ^41^, ^42^, ^43^, ^44^, ^45^, ^46^, ^47^, ^48^, ^49^, ^50^, ^51^, ^52^, ^53^, ^54^, ^55^, ^56^, ^57^, ^58^ ^]^ D) Digital photograph of a scaled‐up SCM_‐20_ labelled with five regions (L1‐L5). E) *I–V* characteristics of the scaled‐up membranes under a 50‐fold NaCl concentration gradient, showing consistent performance across the five regions (L1‐L5).

To demonstrate the superiority of the SCS strategy for SCM fabrication, we first prepared membranes using traditional mixing methods such as physical blending (referred to as TM‐SCM_‐20_). However, for TM‐SCM_‐20_, the weak interaction between the polymer and TpPa‐SO_3_H made film cannot formation. Furthermore, due to the complex ionic matrix of natural seawater, there are inherent differences when simulating its components using only artificial seawater. Hence, to evaluate the performance of SCM in real‐world application scenarios, we assessed the practical applicability of the membrane using seawater collected directly from the ocean. **Figure** **5**A,B illustrate the relationship between membrane structure and energy conversion efficiency in SCM_‐20_ remained at 14.84 W⋅m^−2^ when tested in natural seawater containing complex ionic compositions. Compared with previously reported works (Figure 5C),^[^ ^24^, ^28^, ^29^, ^30^, ^31^, ^32^, ^33^, ^34^, ^35^, ^36^, ^37^, ^38^, ^39^, ^40^, ^41^, ^42^, ^43^, ^44^, ^45^, ^46^, ^47^, ^48^, ^49^, ^50^, ^51^, ^52^, ^53^, ^54^, ^55^, ^56^, ^57^, ^58^ ^]^ where the *X*‐axis represents power density at a 50‐fold salinity difference and the *Y*‐axis represents power density at a 500‐fold salinity difference, SCM_‐20_ ranks among those with the highest reported energy conversion efficiencies, as indicated by the dotted line. The comparison highlights the capacity of SCM_‐20_ to effectively harness energy from higher salinity differences, such as those between concentrated streams from desalination plants and nearby river water. Beyond its energy conversion efficiencies, SCM_‐20_ also features other advantages, including high power conversion stability (Figure 3F) and excellent processibility. To demonstrate the scalability of our SCM fabrication process, we employed a casting machine to produce a large‐scale SCM membrane with dimensions of 150 × 300 mm SCM (Figure 5D). This space‐confined synthesis method offers significant cost advantages through minimized material waste and reduced processing steps compared to conventional layer‐by‐layer fabrication approaches, while maintaining excellent membrane uniformity and performance consistency across the entire area. We assessed the performance across five regions of the membrane, labeled L1‐L5. The consistent separation performance in each region, as depicted by the identical *I–V* curves (Figure 5E), indicates that the SCS strategy can produce a uniform membrane, making it readily scalable for commercial applications.

## 3 Conclusion

We have developed a novel space‐confined synthesis strategy to fabricate a sulfonated covalent organic framework network within a sulfonated polymer matrix, yielding interconnected and well‐defined ion channels with high cation selectivity. The resulting membrane demonstrates surface‐charge‐regulated ion transport characteristics and significantly enhances transmembrane ionic diffusion, effectively reducing ion polarization phenomena. This breakthrough leads to a high power density, placing it in the top tier of reported osmotic energy converters. Our work highlights the potential of the space‐confined synthesis strategy in developing flexible and readily scalable nanofluidic membranes, paving the way for the efficient and sustainable harnessing of blue energy in the global pursuit of clean and renewable energy solutions.

## Conflict of Interest

The authors declare no conflict of interest.

## Supporting information

Supporting Information

## Data Availability

The data that support the findings of this study are available from the corresponding author upon reasonable request.
