# Supplementary material for: Space‐Confined Synthesis of Sulfonated Covalent Organic Framework‒Polymer Membranes for Enhanced Osmotic Energy Conversion
Source: Small. 2025 Aug 25;21(40):e08217. doi: 10.1002/smll.202508217 (PMC12508698; doi:10.1002/smll.202508217)
Supplement: Supplementary file 1 — Supporting Information [file SMLL-21-e08217-s001.pdf]

NANO · MICRO
small

Supporting Information

for *Small*, DOI 10.1002/smll.202508217

Space-Confined Synthesis of Sulfonated Covalent Organic Framework–Polymer Membranes for Enhanced Osmotic Energy Conversion

Yumeng Guo, Xiang Sun, Qianxi Zhang, Ze-Xian Low, Huanting Wang*, Ying Zhu* and Lei Jiang*

Space-Confined Synthesis of Sulfonated Covalent Organic Framework–Polymer Membranes for Enhanced Osmotic Energy Conversion

Yumeng Guo[a, b], *Xiang Sun*[c], *Qianxi Zhang*[a], *Ze-Xian Low*[a, b]*, *Huanting Wang*[b] *, *Ying Zhu*[c d] *, *Lei Jiang*[b, c, d, e]

[a]State Key Laboratory of Materials-Oriented Chemical Engineering, National Engineering Research Center for Special Separation Membrane, Nanjing Tech University, Nanjing, China.

[b]Department of Chemical and Biological Engineering, Monash University, Clayton, Victoria 3800, Australia.

[c]Key Laboratory of Bio-Inspired Smart Interfacial Science and Technology Ministry of Education, School of Chemistry, Beihang University, Beijing 100191, China.

[d]Beijing Advanced Innovation Center for Biomedical Engineering, Beihang University, Beijing 100191, China.

[e]CAS Key Laboratory of Bio-inspired Materials and Interfacial Science, Technical Institute of Physics and Chemistry, Chinese Academy of Sciences, Beijing 100190, China.

***Corresponding authors:** nicholas.low@njtech.edu.cn; huanting.wang@monash.edu; zhuying@buaa.edu.cn

1. Experimental Section/Methods

1.1. Materials

1,3,5-Triformylphloroglucinol (Tp) was purchased from Jilin Chinese Academy of Sciences-Yanshen Technology Co., Ltd. 2,5-Diaminobenzenesulfonic acid (Pa-SO_3H), dimethyl sulfoxide (DMSO), p-Toluenesulfonic acid (PTSA), and sulfuric acid (98 wt%) were supplied by Macklin Chemical Reagent Co., Ltd. (China). Analytical-grade salts (NaCl, KCl, LiCl, $CaCl_2$, $MgCl_2$, $AlCl_3$) and acid/base reagents (HCl, NaOH) were also purchased from Macklin. PEEK was produced by Changchun Jilin University Special Plastic Engineering Research Co., Ltd. (China). Deionized water (18.2 MΩ·cm) was produced using a Milli-Q Reference ultrapure water system.

1.2. Preparation of SPEEK Solution

Poly(ether ether ketone) (PEEK, 7 g) was gradually dissolved in concentrated sulfuric acid (98 wt%, 125 mL) with vigorous mechanical stirring at room temperature. After complete dissolution (typically requiring X hours), the reaction temperature was elevated to 50°C and maintained for 4 hours, during which the solution color transitioned to brown, indicating successful sulfonation. The resulting polymer solution was then precipitated into ice-cold deionized water (approximately 500 mL) to yield light pink sulfonated polymer fibers. These fibers were thoroughly washed with deionized water until neutral pH (pH = 7) was achieved, followed by drying at 60°C in a vacuum oven for 24 hours. ^{1}H NMR analysis confirmed a degree of sulfonation of 75.5% (calculation method described in Supporting Information). For membrane preparation, the obtained SPEEK (2 g) was dissolved in dimethyl sulfoxide (DMSO, 8 g) to prepare a 20 wt% casting solution. The solution was degassed by standing undisturbed for 24 hours at room temperature to eliminate air bubbles prior to membrane fabrication.

1.3. Fabrication of SPEEK and SPEEK/COF Membranes

0.206 g of PTSA monomer and 0.0458 g of Pa-SO_3H monomer were mixed and ground thoroughly to ensure even distribution of the monomers. Then, 0.0342 g of Tp monomer was added to the mixture and ground for an additional 10 min to introduce the Tp monomers and promote interactions between the different COF monomers. Subsequently, 1 ml of DMSO and 2 g of 20% SPEEK casting solution were added to the mixture, followed by grinding for another 10 min. The resulting mixture was evenly coated onto a clean glass substrate using a manually adjusted Doctor's blade to control the film thickness, resulting in a uniform and flat film. The

coated film was then heated at 80°C for 72 h. This step facilitated the solidification and crystallization of the film to form a stable composite structure. The obtained membranes were designated as SCM_{-X} membranes (where X represents the amount of COF). For comparison, pure SPEEK membranes were prepared following the same procedure but without the addition of COF monomers.

1.4. Membrane Surface Analyses

The surface elemental composition of the membranes was characterized by X-ray photoelectron spectroscopy (XPS, Thermo ESCALAB 250XI). Solid-state ^{13}CNMR spectra were obtained through cross-polarization magic angle spinning (CP-MAS) methodology on an AVANCE III HD 500 MHz NMR spectrometer. Fourier-transform infrared (FTIR) spectral data for the SCM membranes were collected with an Excalibur 3100 spectrometer, and all the data are reported on a wavenumber (cm^{-1}) scale. The CO_2 adsorption isotherms were generated via a Micromeritics ASAP 2460 analyzer, following sample pretreatment involving vacuum degassing ($<10^{-2}$ Pa) at 120°C for 24 h and subsequent measurement at 77 K maintained by iced water cooling. Surface hydrophilicity was evaluated using an OCA40Micro contact angle system (Dataphysics Instruments GmbH), where 2.0 μL aliquots of 0.1 M KCl buffer solutions (pH-adjusted) were dispensed onto membrane surfaces. Scanning electron microscopy (SEM) imaging was conducted on an SU-8010 instrument operated at 5 kV, with samples sputter-coated with gold (SCD 040 Balzers Union) to mitigate charging artifacts. Small-angle X-ray scattering (SAXS) measurements utilized a SAXSpace apparatus (AntonPaar), requiring membrane pretreatment through 24 h immersion in 2 M CsCl solution, extensive water rinsing, and oven-drying at 90 °C for 24 hours. Zeta potential values of both SCM and SPEEK membranes were quantified using an Anton Paar SurPASS analyzer in 1 mM KCl solutions across pH 3.0–11.0. For transmission electron microscopy (TEM) characterization, membrane specimens mounted on copper grids were directly imaged via a Tecnai F20 microscope at 200 kV acceleration voltage. Atomic force microscopy (AFM) topographical profiles in ambient air were acquired using a Bruker Dimension Icon system operating in tapping mode.

1.5. Electrical Measurement

The membranes were assembled into a testing cell positioned between two electrolyte chambers, with paired Ag/AgCl electrodes. The active membrane area dedicated to energy harvesting was approximately 3×10^{-8} m^2. Open-circuit voltage (V_{OC}) and short-circuit current

(I_{SC}) were derived from current-voltage (*I-V*) curve measurements under imposed transmembrane concentration gradients. Voltage scanning was performed linearly from −0.2 to +0.2 V at 0.02 V intervals. In this system, V_{OC}, E_{redox}, E_{diff}, and R_o correspond to the experimentally recorded potential, interfacial redox potential disparity at electrode-electrolyte junctions, ion-selective membrane-induced diffusion potential, and membrane internal resistance, respectively. Specifically, V_{OC} is a composite parameter expressed as $V_{OC} = E_{diff} + E_{redox}$. To experimentally isolate E_{redox}, the selective membrane was replaced with a non-selective silicon membrane featuring a single microwindow, thereby eliminating E_{diff}. Under a 50-fold NaCl concentration gradient, the measured E_{redox} reached 42 mV. Electrode potentials exhibited negligible drift during calibration owing to minimal bulk concentration changes within the initial minutes of ion diffusion. For a specified concentration gradient, the cationic transference number (t_+) is determined by the following expression:

$$t_+ = \frac{1}{2}\left(\frac{E_{diff}}{\frac{RT}{zF}\ln\frac{\gamma_{c_H} c_H}{\gamma_{c_L} c_L}} + 1\right)$$

where R (gas constant), T (absolute temperature), z (ionic charge valence), F (Faraday constant), γ_{cH} and γ_{cL} (activity coefficients), and c_H, c_L (high and low ion concentrations) denote the thermodynamic and electrochemical parameters governing the system.

1.6. Computational Simulations

Molecular dynamics (MD) simulations were applied to investigate Na^+ diffusion in aqueous NaCl solutions on SPEEK and SCM. The solid–liquid interface calculations were conducted with COMPASS III force field parameters using the Forcite module in MS 2020. The SPEEK membrane contained 15 SPEEK chains with dimensions of 37.56 Å×37.56 Å×29.99 Å, and the SCM was composed of the same number of SPEEK chains as well as 3 TpPa-SO_3H flakes with a size of 41.05 Å×41.05 Å×29.99 Å. The NaCl aqueous solution contained 40 NaCl and 2222 H_2O molecules. After geometry optimization, the aqueous solution was placed on the optimized membrane surface. Molecular dynamics (MD) simulations were conducted within the canonical (NVT) ensemble at 298.0 K, utilizing a temporal resolution of 1 fs and cumulative simulation duration of 800 ps. Trajectory data were sampled at 8 ps intervals throughout the simulation period, ensuring sufficient duration for system energy and thermal equilibrium stabilization. Temperature regulation was achieved via a Nose-Hoover thermostat. Electrostatic interactions were computed using the Ewald

summation method, while van der Waals (vdW) forces were approximated through an atom-based cutoff scheme with a 12.5 Å spherical radius.

2. Supplementary Figures

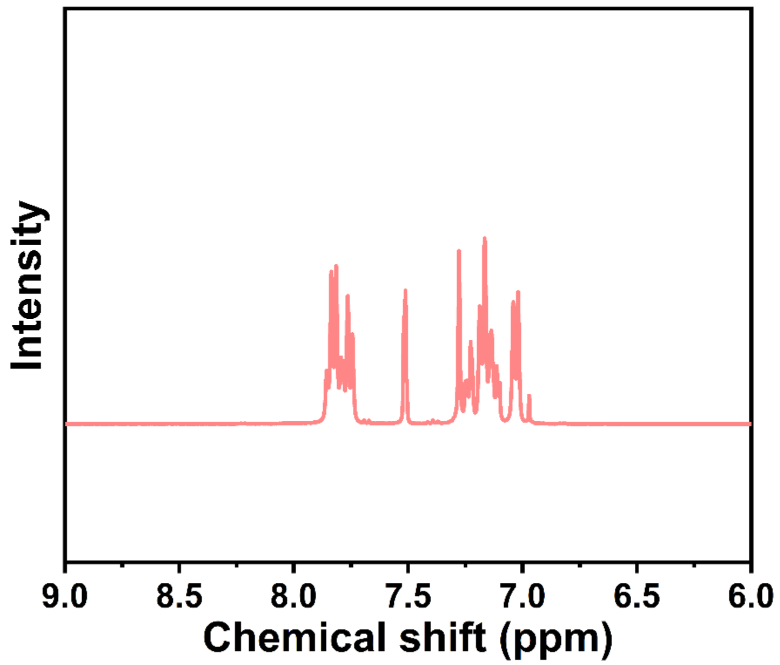

Figure S1. ^{1}H NMR spectrum of the SPEEK with 4h sulfonation time (From the spectrum, the degrees of sulfonation (DS) of the obtained SPEEK were calculated to be 75.5%).

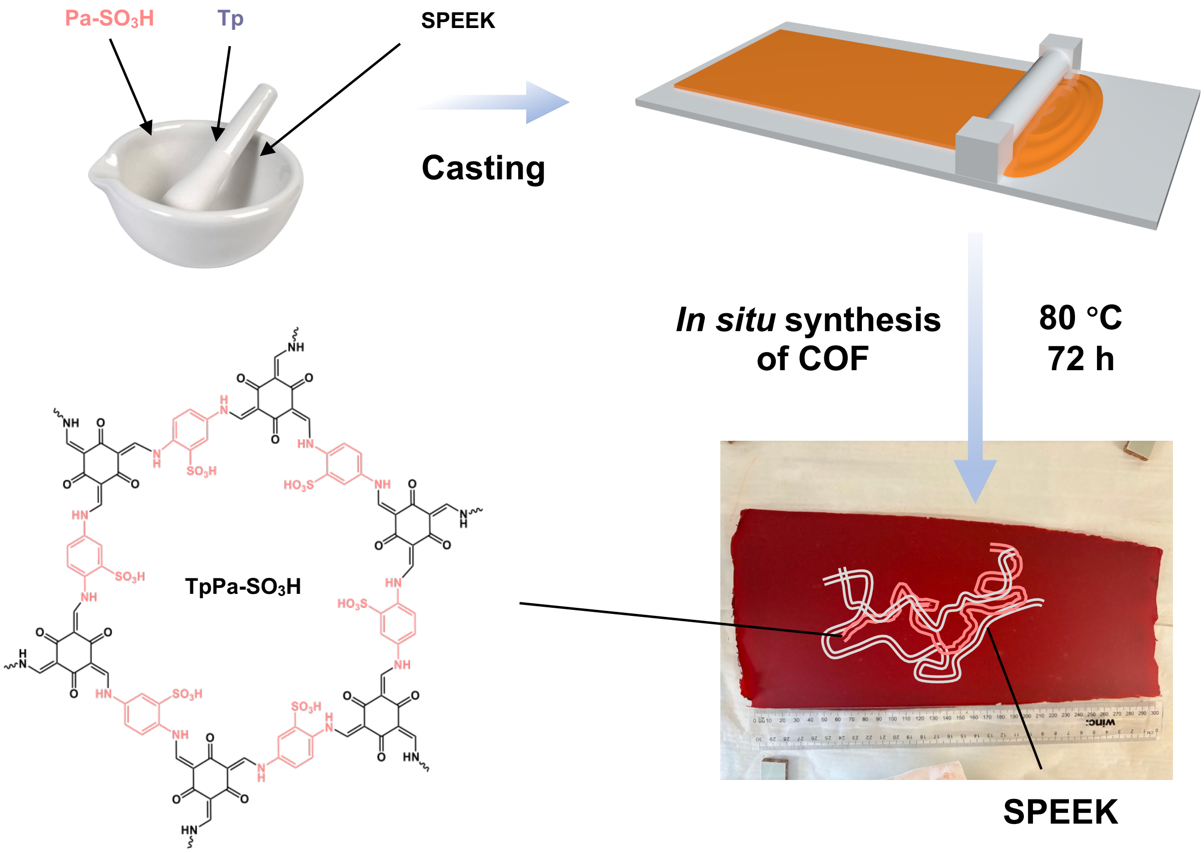

Figure S2. Schematic diagram of the preparations of the SCM. Mixture of TpPa-SO_3H monomer and SPEEK was ground for 12 h, which was blade-coated onto a glass with a 100 μm thick scraper. Membrane was heated to 80 °C for 72 h and peeled off from the glass to obtain the self-standing SCM.

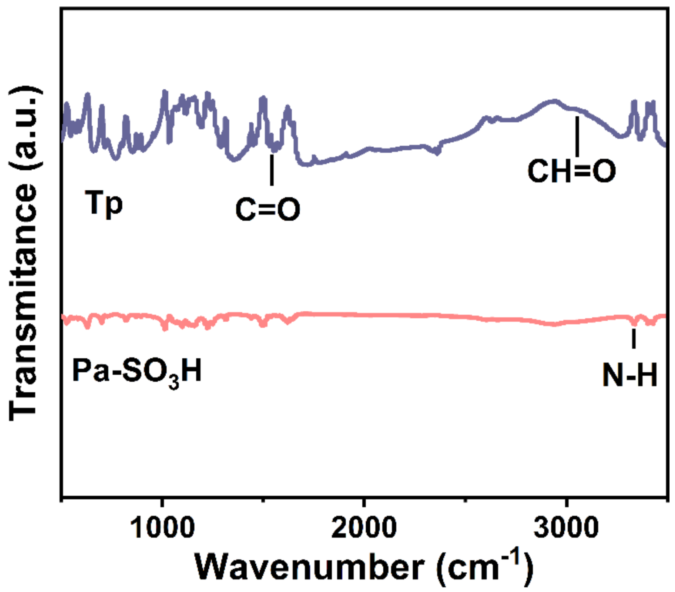

Figure S3. FTIR spectra of Tp and $Pa\text{-}SO_3H$ monomer.

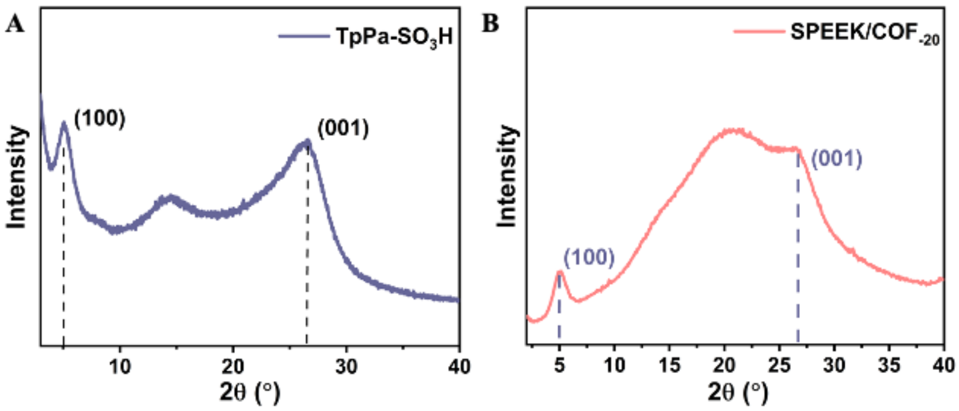

Figure S4. XRD pattern of (**A**) TpPa-SO_3H and (**B**) SCM_{-20}.

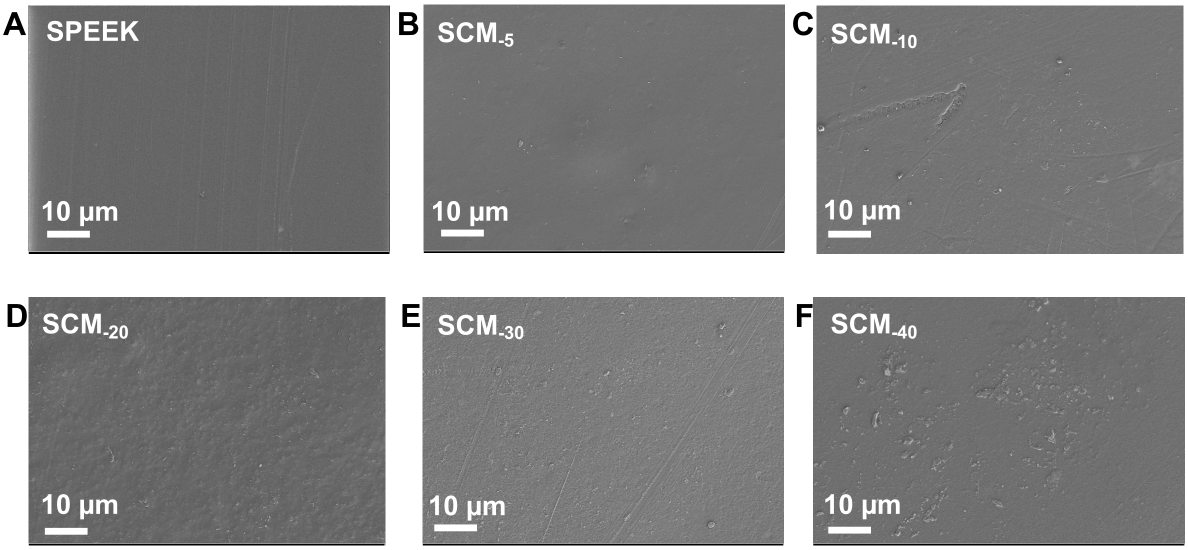

Figure S5. SEM surface images of (**A**) SPEEK, (**B**) SPEEK/COF$_{-5}$, (**C**) SPEEK/COF$_{-10}$, (**D**) SPEEK/COF$_{-20}$, (**E**) SPEEK/COF$_{-30}$, (**F**) SPEEK/COF$_{-40}$.

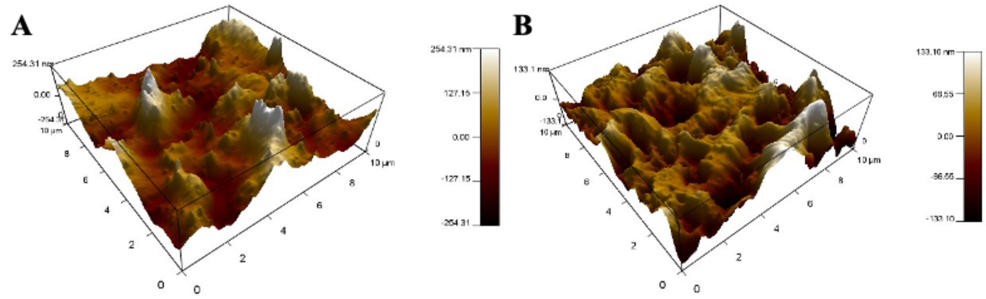

Figure S6. The AFM images of front (**A**) and back (**B**) surfaces of SCM$_{-20}$.

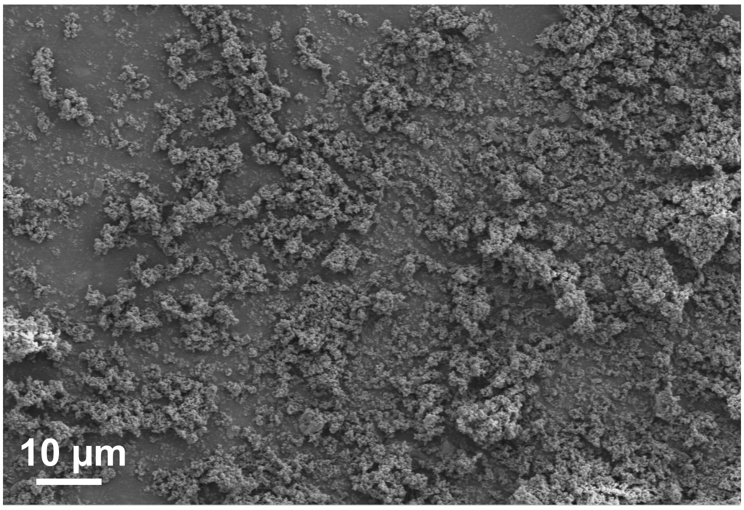

Figure S7. Morphology of recovered TpPa-SO_3H from re-dissolved SCM.

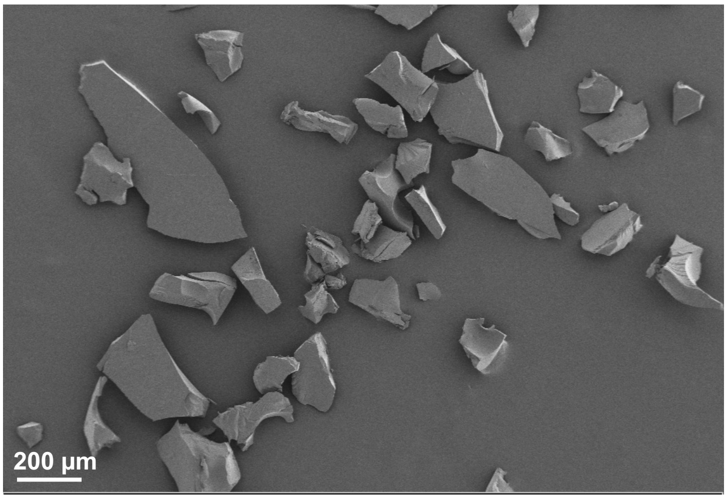

Figure S8. Morphology of directly synthesized TpPa-SO_3H.

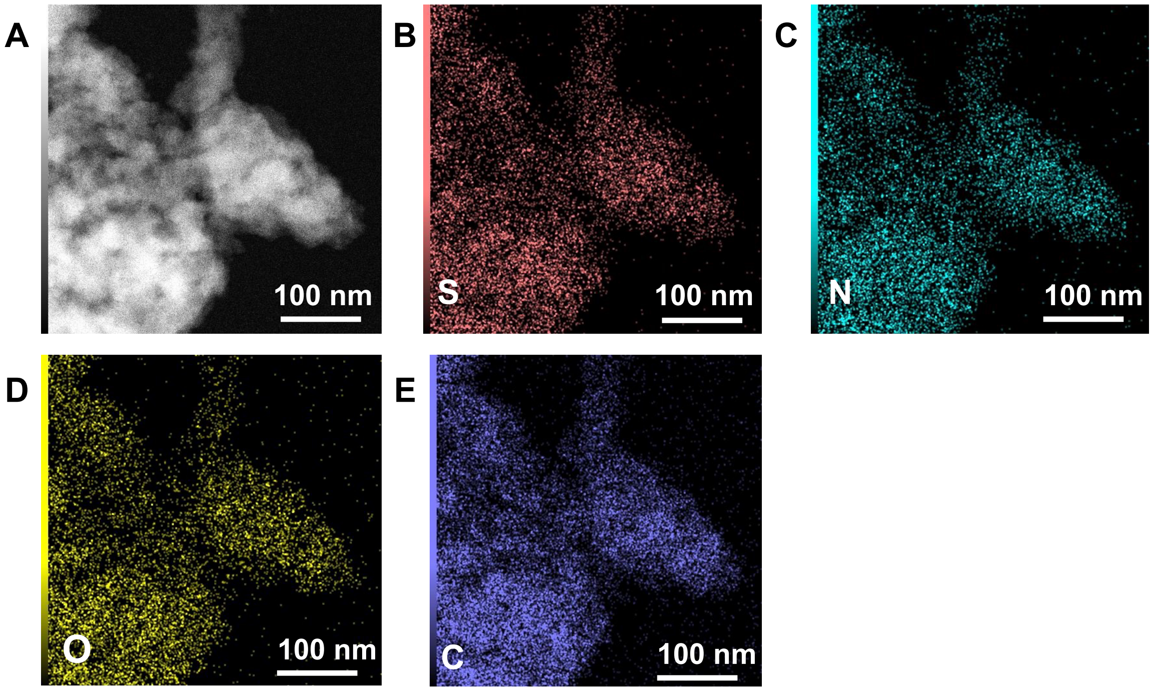

Figure S9. (A) TEM image: The surface of SCM$_{-20}$. (B-E) EDS mapping of SCM$_{-20}$.

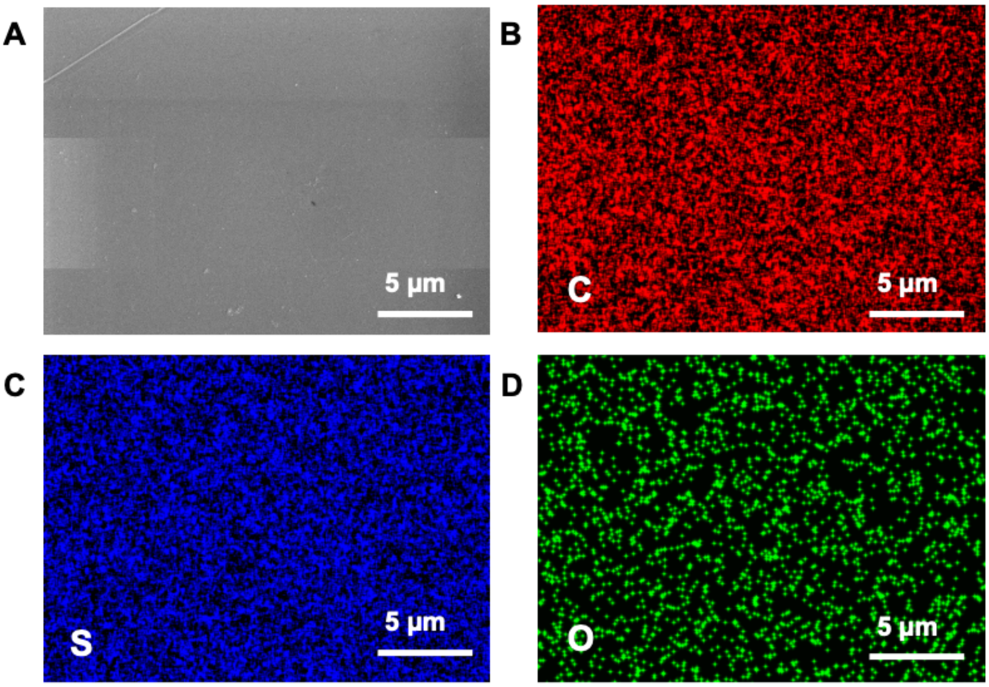

Figure S10. (**A**) SEM image of the surface of SPEEK membrane. (**B-D**) EDS mapping of SPEEK membrane.

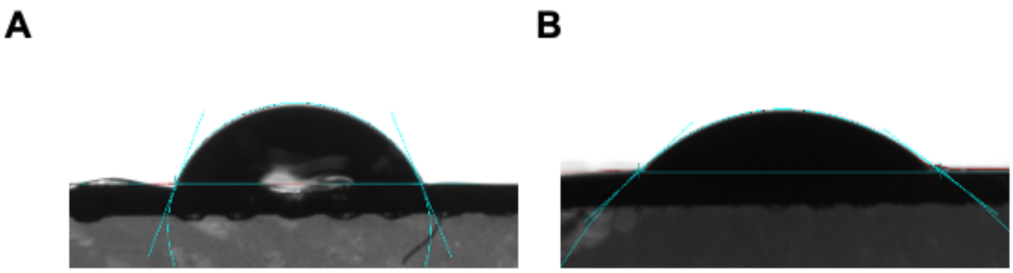

Figure S11. Static contact angle photo of (**A**) SPEEK membrane; (**B**) SPEEK/COF$_{-20}$ composite membrane.

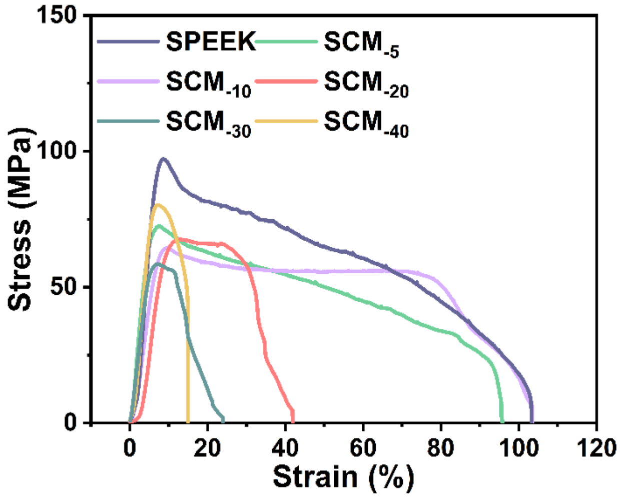

Figure S12. Fracture tensile stress–strain curves of various SCM. Mechanical integrity plays a crucial role in the fabrication of polymer membranes for practical applications. The SCM exhibited excellent strain resistance, which was attributed to the skeleton of TpPa-SO_3H being a rigid material. Moreover, the introduction of TpPa-SO_3H reduced the strain of the SCM from 103% to 15%.

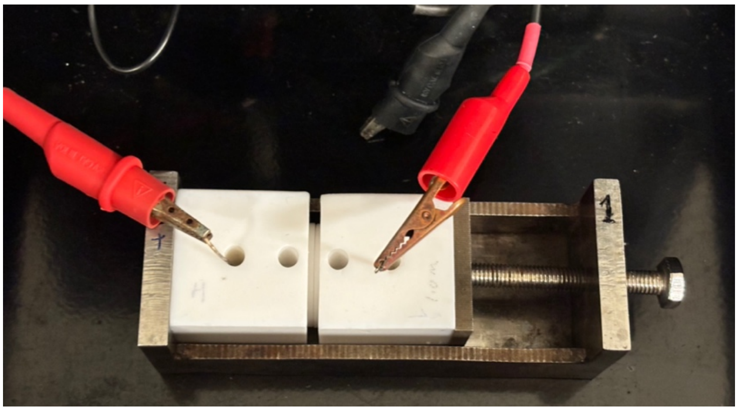

Figure S13. Photograph of the electrical measurement device.

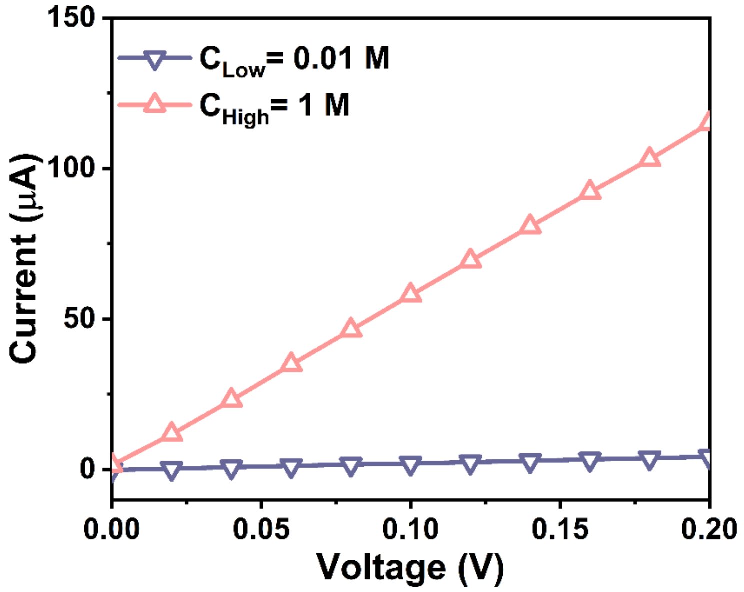

Figure S14. Ion selectivity test of SCM_{-20} based on the electrochemical method.

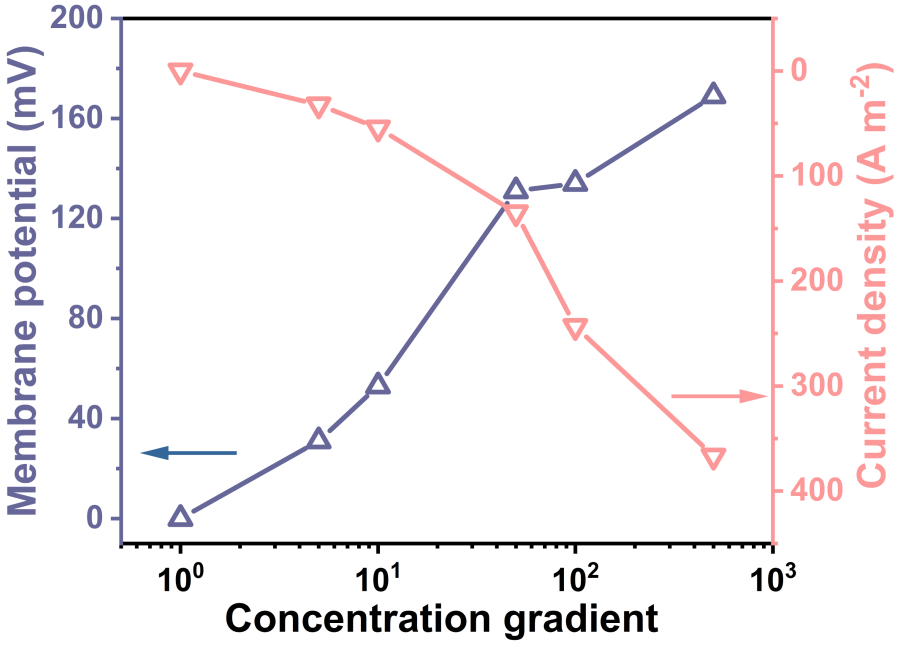

Figure S15. I_{SC} and V_{OC} of SCM$_{-20}$ under different concentration gradients.

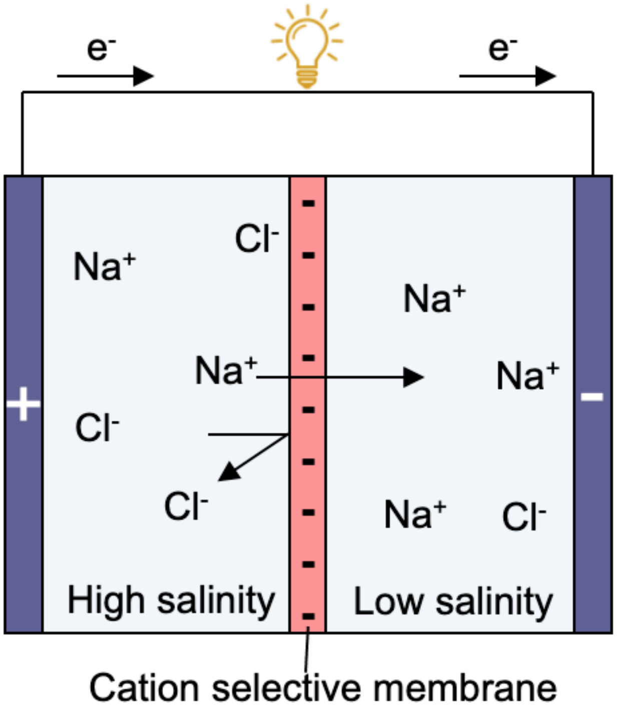

Figure S16. Illustration of salinity gradient conversion.

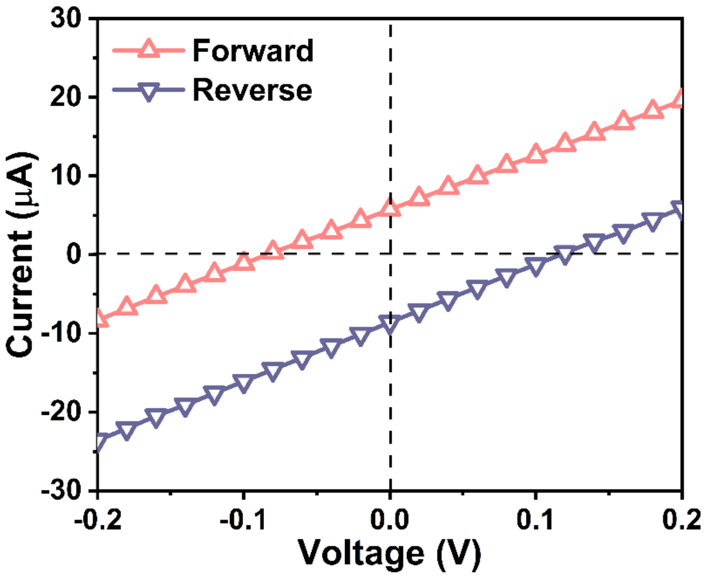

Figure S17. Forward and reverse diffusion *I*-*V* curves for the SCM$_{-20}$ tested in a 50-fold NaCl concentration.

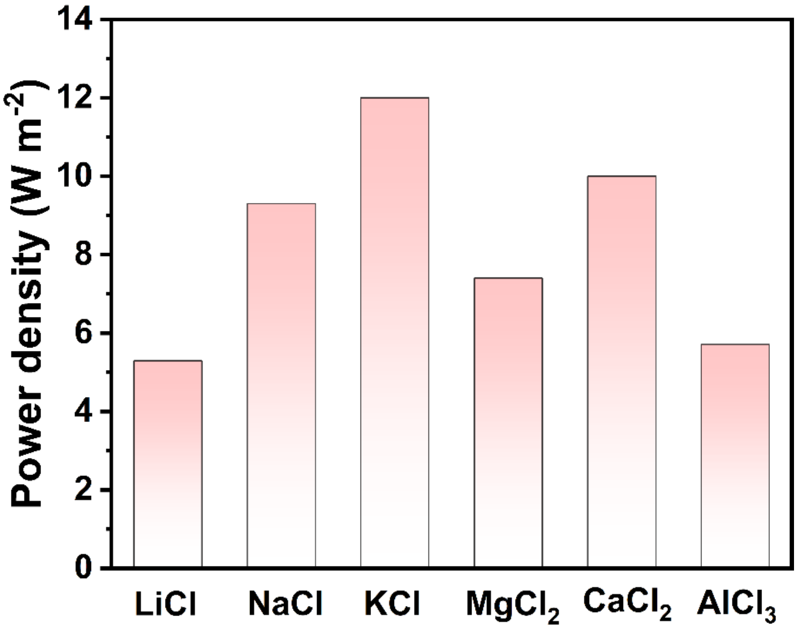

Figure S18. Power density generation of SCM-20 under a series of 50-fold electrolyte species.

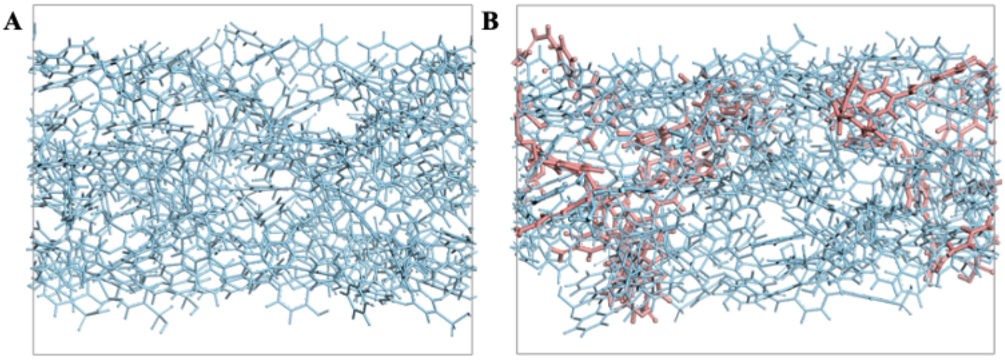

Figure S19. Simulated SPEEK (**A**) and (**B**) SCM_{-20} membrane structures.
